# The Effect of Resveratrol on Mitochondrial Function in Myoblasts of Patients with the Common m.3243A>G Mutation

**DOI:** 10.3390/biom10081103

**Published:** 2020-07-24

**Authors:** Leila Motlagh Scholle, Helena Schieffers, Samiya Al-Robaiy, Annemarie Thaele, Faramarz Dehghani, Diana Lehmann Urban, Stephan Zierz

**Affiliations:** 1 Department of Neurology, Martin-Luther-University Halle-Wittenberg, 06120 Halle, Germany; helena@schieffers.de (H.S.); annemarie.thaele@uk-halle.de (A.T.); stephan.zierz@uk-halle.de (S.Z.); 2 Center for Basic Medical Research, Martin-Luther-University Halle-Wittenberg, 06120 Halle, Germany; samiya.al-robaiy@uk-halle.de; 3 Department of Anatomy and Cell Biology, Martin Luther University Halle-Wittenberg, 06108 Halle, Germany; faramarz.dehghani@medizin.uni-halle.de; 4 Department of Neurology, Ulm University, 89070 Ulm, Germany; diana.lehmann@rku.de

**Keywords:** resveratrol, m.3243A>G mutation, SIRT1, SIRT3, OXPHOS

## Abstract

Mitochondrial function is essential for ATP-supply, especially in response to different cellular stressors. Increased mitochondrial biogenesis resulting from caloric restriction (CR) has been reported. Resveratrol (RSV) is believed to mimic the physiological effects of CR mainly via a sirtuin (SIRT) 1-dependent pathway. The effect of RSV on the physiological function of mitochondrial respiratory complexes was evaluated using a Seahorse XF96. Myoblasts of five patients harboring the m.3243A>G mutation and five controls were analyzed. The relative expression of several genes involved in mitochondrial biogenesis was evaluated for a better understanding of the coherent mechanisms. Additionally, media-dependent effects of nutritional compounds and hormonal restrictions (R) on myoblasts from patients and controls in the presence or absence of RSV were investigated. Culturing of myoblasts under these conditions led to an upregulation of almost all the investigated genes compared to normal nutrition. Under normal conditions, there was no positive effect of RSV on mitochondrial respiration in patients and controls. However, under restricted conditions, the respiratory factors measured by Seahorse were improved in the presence of RSV. Further studies are necessary to clarify the involved mechanisms and elucidate the controversial effects of resveratrol on SIRT1 and SIRT3 expression.

## 1. Introduction

Mitochondria are the cells’ main energy sources, converting nutrients into usable energy [1]. The mitochondrial DNA (mtDNA) is a double-stranded 16.5 kb circle molecule, encoding for 13 essential subunits of the mitochondrial respiratory chain unit, two ribosomal mt-RNAs (rRNAs) and 22 mitochondrial transfer RNA (mt-tRNA)s [2,3]. Mitochondrial diseases can either be caused by mutations in the mtDNA itself or by mutations of nuclear origin and are associated with a wide range of different clinical phenotypes, from mild to severe [4]. The coexistence of mutant and wild-type mtDNA molecules within the same cell is defined as heteroplasmy [5]. It is already known that patients with higher heteroplasmy levels tend to have more severe disease burden and progression rate; however, disease burden and progression vary greatly between individuals and tissue [3]. Recently, it has been shown that heteroplasmy levels did not differ between clinically affected and unaffected m.3243 patients [3]. The m.3243A>G point mutation in the *MT-TL1* gene (encoding mt-tRNA^Leu(UUR)^) can be found in approximately 80% of patients with MELAS (mitochondrial encephalopathy, lactate acidosis, and stroke-like episodes)-syndrome [3,6,7,8].

Resveratrol (3,4,5-trihydroxystilbene, RSV) is a small phenolic compound and found in grapes, nuts, berries, and various other plants [9]. During the years 2008–2010, the effect of RSV for the treatment of patients with MELAS-syndrome has been evaluated in a clinical study [10]. For this purpose, the Resveratrol analog SRT501 (Sirtris Pharmaceuticals, Cambridge, MA, USA) was used. RSV has been referred to as the caloric restriction “mimetic” compound [11].

The dual control of mitochondrial biogenesis by sirtuin (SIRT) 1 and SIRT3 is widely believed [12]. SIRT1 activates the peroxisome proliferator-activated receptor Gamma coactivator 1 α (PGC-1α)-mediated transcription of nuclear and mitochondrial genes. PGC-1α is known to be a central inducer of mitochondrial biogenesis [13], and co-activates the transcription of Nuclear Respiratory Factor (NRF) 1, which regulates the transcription of *Tfam*. Mitochondrial transcription factor A (TFAM) stimulates mitochondrial DNA replication and mitochondrial gene expression in the mitochondrial matrix. The regulatory effect of SIRT1 on PGC-1α activity and its role in mitochondrial biogenesis is controversially discussed [14]. Some groups reported the induction of genes for oxidative phosphorylation and mitochondrial biogenesis and an increase of PGC-1α activity by SIRT1 [15]. On the other hand, others opposed the obligatory regulatory role of SIRT1 for the PGC-1α- mediated mitochondrial biogenesis in muscle. They showed the downregulation of *PGC-1α* and *Tfam* resulting from the overexpression of *SIRT1* in muscle and the downregulated levels of *SIRT1* by upregulation of PGC-1α in this tissue [16]. SIRT3 directly activates important proteins for oxidative phosphorylation, tricarboxylic acid (TCA) cycle, and fatty-acid oxidation, and indirectly affects PGC-1α and AMP-activated protein kinase (AMPK) [12].

Nevertheless, the activating effect of RSV on SIRT1 and SIRT3 is a matter of debate. Many studies reported on SIRT1 and SIRT3 activation by RSV and their structurally related compounds [17,18]. Others, however, denied RSV and its analogs as direct SIRT1-activators [19]. In a zebrafish-model, RSV did not affect the mRNA level of *SIRT1* and *PGC-1α* and even decreased the expression of *SIRT3* and *SIRT4* genes [20].

**Figure 1:**
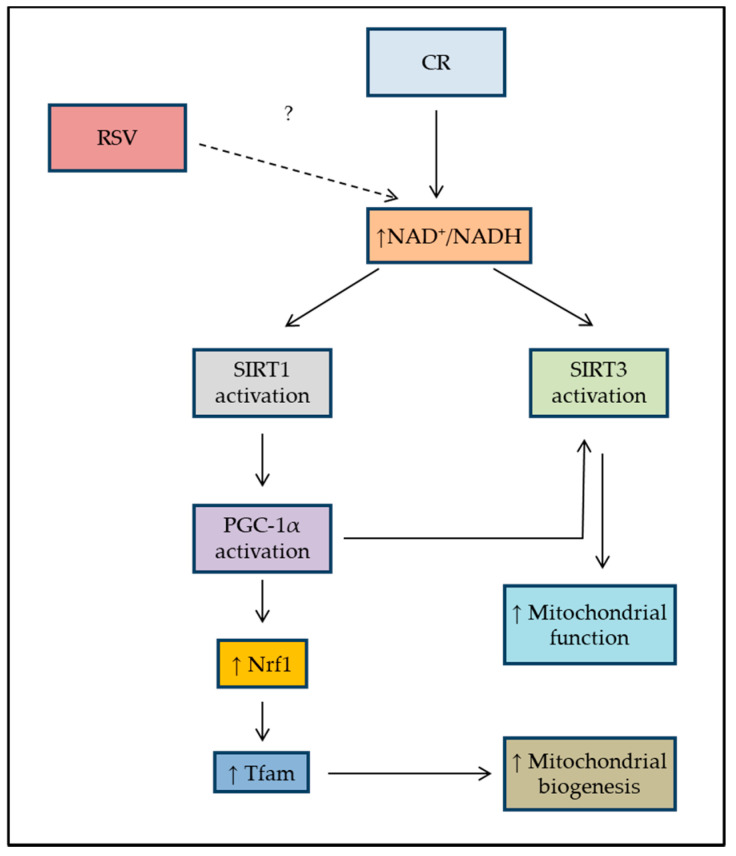
Schematic diagram showing the investigated pathways in the present study using RSV in patients and controls, adopted accordingly [21]. Caloric restriction (CR) activates the SIRT1 levels or NAD^+^ levels leading to the activation of PGC-1α in the nucleus, which then activates the transcription of genes that are necessary for mitochondrial function and biogenesis. CR also leads to activation of AMPK and, therefore, the activation of PGC-1α in skeletal muscle. RSV: Resveratrol; NAD: Nicotinamide adenine dinucleotide; SIRT1: sirtuin 1; SIRT3: sirtuin 3; PGC-1α: peroxisome proliferator-activated receptor gamma co-activator 1α; Nrf1: Nuclear Respiratory Factor 1; Tfam: Mitochondrial Transcription Factor A.

The aim of this study was to assess the effect of RSV on oxidative phosphorylation in patients harboring the m.3243A>G mutation and in controls. The controversially discussed caloric restriction (CR) stimulating effect of RSV on mitochondrial respiratory activity and mitochondrial biogenesis was evaluated in patients and in controls under normal and restricted conditions. The investigated pathway is schematically shown in Figure 1. The potential protective effects of RSV were only investigated under restricted cultural conditions to comply with the basic cellular needs, as well.

## 2. Materials and Methods

### 2.1. Human Myoblasts

Muscle primary cells from five patients harboring the genetically confirmed m.3243A>G mutation and controls were provided by the Muscle Tissue Culture Collection (MTCC) from the University of Munich. The presence of a mutation was confirmed in myoblasts of all patients. Further details are given in Table 1. Five patients served as controls (two males, three females), who had muscle biopsy for the diagnosis of a suspected neuromuscular disorder. They were deemed to be ‘normal controls’ if they were ultimately found to have no muscle disease by combined clinical and histologic criteria. The age of the controls ranged from 35 to 53 years.

#### Myoblast Culture Conditions

The experiments were divided into two main groups depending on the culture conditions of the myoblasts: (I) normal (N)- or, (II) substrate restricted (R)-conditions, both, either without or with 10 µM or 20 µM of RSV. At first, all cells were grown in skeletal muscle cell growth medium (Promocell, Heidelberg, Germany) supplemented with 10% fetal bovine serum (FBS), GlutaMAX-1 (Gibco, Life Technologies, Grand Island, NY, USA), and Supplement mix (Fetuin (bovine, 50 ng/mL), human epidermal growth factor (hEGF, 10 pg/mL), human basic fibroblast growth factor (hbFGF, 1 pg/mL), Dexamethasone (0.4 pg/mL), and human recombinant insulin (10 ng/mL), Promocell, Heidelberg, Germany). After the first 24 h, the medium was changed and the cells were cultured for another 48 h. In the R group, the medium was replaced by a substrate-limited medium (DMEM with 0.5 mM glucose, 1.0 mM glutamine, and 1% FBS) with or without 10 µM or 20 µM RSV. Promocell skeletal muscle cell growth medium was used either without or with 10 µM or 20 µM RSV in the normal group. Resveratrol (>99% purity) was obtained from Sigma-Aldrich (St. Louis, MO, USA). All cells were maintained in 5% CO_2_ at 37 °C.

### 2.2. The Seahorse XF96 Analysis of Metabolic Function

To evaluate the mitochondrial function and the effect of RSV in patients and controls, the Mito Stress test was performed using a Seahorse XF96 Cell Analyzer (Seahorse Bioscience, Billerica, MA, USA), either under N or restricted R conditions according to manufacturer’s recommendations. Briefly, myoblasts from patients and controls were seeded to Seahorse XF96 cell culture microplates (2.5 × 10^4^ cells per well) in skeletal muscle cell growth medium supplemented with 10% FBS. After a 24-hour incubation at 37 °C, the medium was replaced depending on six different experimental conditions, as described in Section 2.1 (R or N cultural conditions without or either with 10 µM or 20 µM RSV).

Forty-eight h later the cells were washed twice with the pre-warmed assay medium (XF base medium supplemented with 10 mM glucose, 2 mM glutamine, and 1 mM sodium pyruvate; pH 7.4).

Oxygen consumption rate (OCR) values were measured following sequential injections of oligomycin (2 µM), carbonyl cyanide p-trifluoromethoxyphenylhydrazone (FCCP, 2 µM), and rotenone (0.5 µM) + antimycin A (0.5 µM), with three OCR measurements after each injection following an injection of cell-permeable Hoechst 33342 (2 µg/mL) dye. The key parameters of mitochondrial function such as basal respiration (BR), ATP-linked respiration, maximal respiration (MR), and spare respiratory (SRC) capacity were analyzed using the above-described measurements. The ATP linked respiration (ATP production rate, ATP-R) was derived from the difference between the OCR at baseline and respiration following oligomycin addition. Maximal OCR was determined by subtracting the OCR after antimycin A addition from the OCR induced by FCCP. The SRC was calculated by the difference between maximal and basal respiration. The data were normalized to cell numbers by measurement of Hoechst dye staining of nuclei with excitation and emission wavelengths 355 nm and 465 nm, accordingly, using a Tecan Infinite^TM^ M1000 (Tecan, Groedig, Austria) and plotted as OCR (pmol/min/cell ± SD).

### 2.3. Gene Expression by Quantitative Real-Time (qRT)-PCR

The cells from the six different groups, as described in Section 2.1 (N or R with or without 10 µM or 20 µM RSV), were harvested, shock frozen in liquid nitrogen, and stored at −80 °C until RNA extraction. RNA was extracted using a NucleoSpin RNA kit (Macherey and Nagel, Duren, Germany), according to the manufacturer’s instructions. cDNA was next synthesized using the reverse transcription of 1 µg of RNA with RevertAid H Minus First Strand cDNA Synthesis kit (Thermo Scientific, Vilnius, Lithuania), according to the manufacturer’s instructions. cDNAs were kept at −20 °C until analysis.

Quantitative Real-Time (qRT)-PCR was carried out using PowerUp™ SYBR™ Green Master Mix (Thermo Scientific, Vilnius, Lithuania) using a QuantStudio 3 real-time PCR machine (Applied Biosystems, Thermo Fisher, Foster City, CA, USA). Each 10 µL-reaction contained 5 µL (2×) SYBR Green master mix, 500 nM forward and reverse primer, 0.5 µL cDNA, and nuclease-free water. The used primer pairs are listed in Table 2. The following thermal program was applied: a single cycle of DNA polymerase activation for 15 min at 95 °C followed by 40 amplification cycles of 15 s at 95 °C (denaturation) and 1 min at 60 °C (annealing and extension). Subsequently, a melting temperature analysis of the amplification products was performed by gradually increasing the temperature from 60 to 95 °C in 15 min. The fluorescent reporter signal was normalized against the internal reference dye (ROX) signal. The relative gene expression (ΔΔCT) was calculated first by correcting each gene cycle threshold (CT) by the average CT value for the housekeeping genes *HPRT1* and *β-Actin,* that were stable across groups (calculation of relative expression—reported as 2^−ΔΔCt^ and CT representing the cycle threshold). Three technical replicates were measured for each sample in three independent experiments.

### 2.4. Statistical Analysis

Statistical analysis, calculation, and visualization were performed using Prism 8 (GraphPad, San Diego, CA, USA). An analysis of correlation was carried out using a two-way analysis of variance (ANOVA) followed by Tukey’s post hoc test. The level of significance was set to *p* = 0.05. The statistical tests chosen were predetermined by the size of the study group and the numerical range of values.

### 2.5. Ethical Statement

The study was conducted in accordance with the Declaration of Helsinki and was approved by the local Ethics Committee of the University Halle-Wittenberg (Project identification codes 215/20.01.10/3 and 2020-019). A written informed consent was received from all patients.

## 3. Results

For better readability of the results, the experiments using 48 h cultures were divided between the ones conducted either under normal (N) or restricted (R) conditions.

### 3.1. The Seahorse XF96 Analysis of Metabolic Function

#### 3.1.1. The Effect of Restricted Conditions

**Figure 2:**
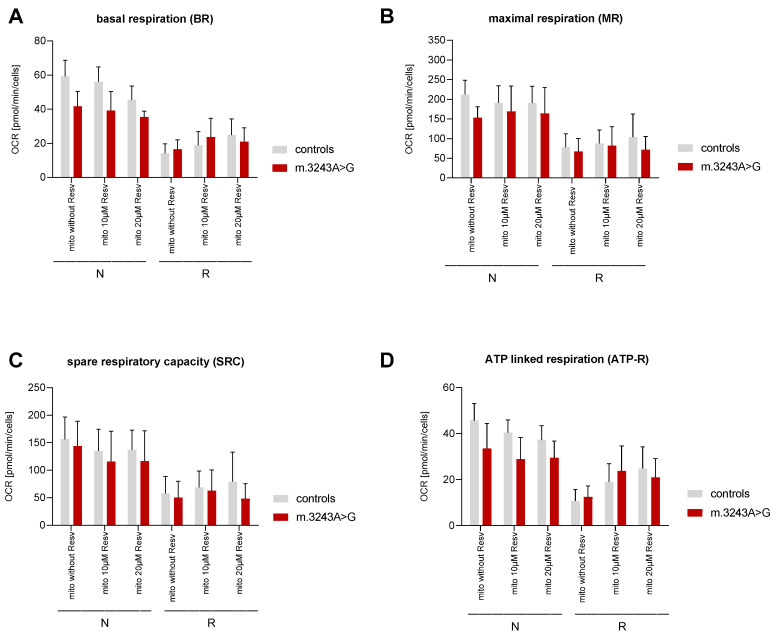
Evaluation of mitochondrial function using a Seahorse XF96 Cell Analyzer in myoblasts from patients (n = 5) and controls (n = 5). The key parameters of mitochondrial function such as basal respiration (BR), ATP production (ATP-R) and spare respiratory capacity (SRC) were analyzed as previously described. (**A**) Basal respiration (BR), (**B**) maximal respiration (MR), (**C**) spare respiratory capacity (SRC) and (**D**) ATP-linked respiration (ATP-R) after 48 h under normal (N) and restricted (R) conditions. The significant differences are shown in Table 3 and Table 4, and Table S1.

Independent of RSV-absence or presence, restriction in the culture medium led to a decrease of oxidative phosphorylation (OXPHOS) factors (Figure 2). This decrease was significant, except in one case—the decrease of ATP production in the presence of 10 µM RSV was only significant in controls.

The mean values are presented in Supplementary Table S1 (*p* values are only shown in case of significance).

#### 3.1.2. Differences between Patients Harboring the m.3243A>G Mutation and Controls

##### Mito Stress Test in the Absence of Resveratrol

Under normal (N) conditions, BR, MR, and ATP-R were all significantly higher in controls compared to patients without the addition of RSV (Figure 2 and Table 3). However, under R conditions, no significant differences in the above-mentioned factors were detected between patients and controls.

##### Mito Stress Test in RSV-treated Groups

In experiments under N conditions, BR and ATP production were significantly higher in controls than in patients in the presence of RSV. MR and SRC were similar in patients and controls (Figure 2 and Table 3).

In all R groups, all the above-mentioned values were comparable between patients and controls (Figure 2 and Table 3).

#### 3.1.3. The Effect of RSV on OXPHOS Factors

##### The Effect of RSV on OXPHOS Factors under Normal Conditions

Upon treatment of myoblasts with 10 or 20 µM RSV under N condition, there was no significant difference between values resulting from either 10 or 20 µM RSV in all of the main OXPHOS factors (BR, MR, SRC, and ATP-R) with only one exception. As the only exception, BR was significantly lower in controls in the presence of 20 µM RSV (Figure 2 and Table 4).

##### The Effect of RSV on OXPHOS Factors under Restricted Conditions

The addition of 10 or 20 µM RSV under the R condition led to an improvement of ATP-R in controls and patients (Figure 2 and Table 4).

### 3.2. Gene Expression by qRT-PCR

#### 3.2.1. The Effect of Restrictions

**Figure 3:**
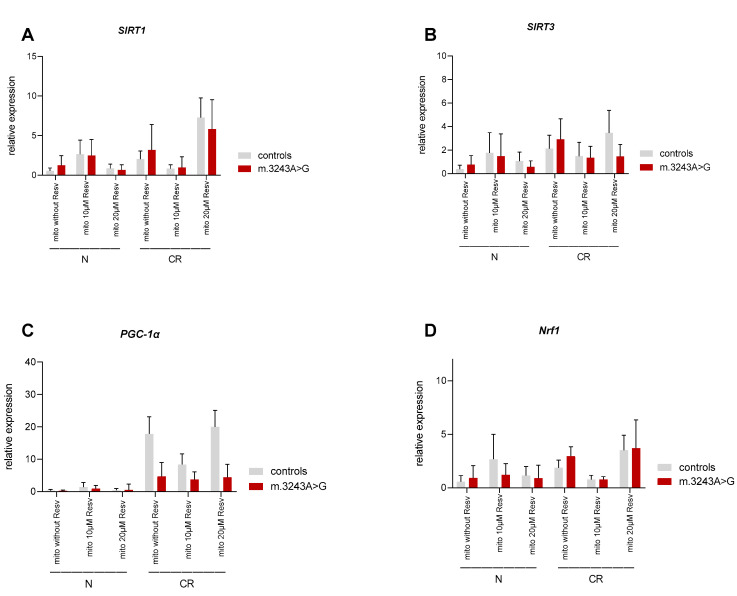

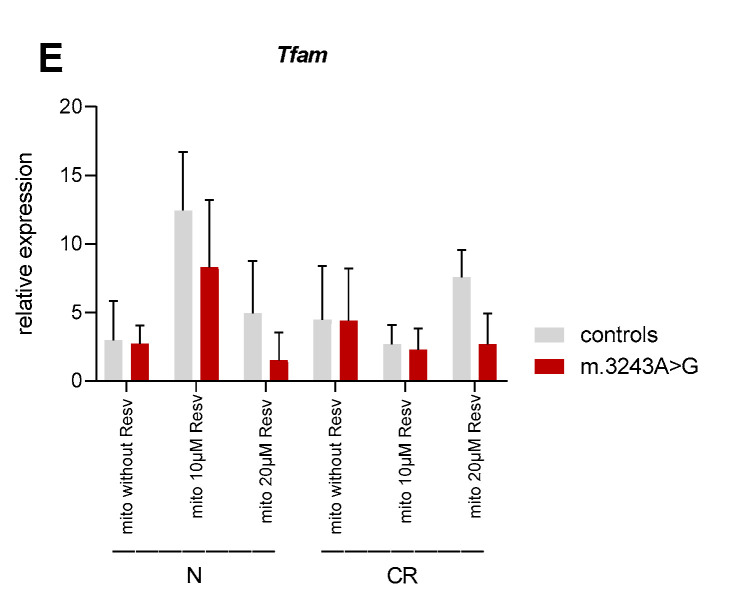
Evaluation of gene expression in myoblasts from patients (n = 5) and controls (n = 5), analyzed as previously described. (**A**) *SIRT1*, (**B**) *SIRT3*, (**C**) *PGC-1 α*, (**D**) *Nrf1*, and (**E**) *Tfam* after 48 h, N vs. R conditions. The significant differences are shown in Table 5 and Table 6, and Table S2.

Under R conditions, the expression of *SIRT1*, *SIRT3*, *PGC-1α*, *Nrf1*, and *Tfam* tended to be increased in the absence of RSV or in the presence of 20 µM RSV compared to normal cultural conditions in both, controls and patients. The expression rates in the presence of 10 µM RSV did not follow any specific pattern (Table 5 and Figure 3).

#### 3.2.2. The Difference between Patients and Controls

Without RSV, there was no significant difference in expression of *SIRT1*, *SIRT3*, *Nrf1*, and *Tfam* between patients and controls under normal and restricted conditions. The expression of *PGC-1 α* was significantly lower in patients compared to controls only in restricted conditions in the absence or presence of RSV (Figure 3). In the majority of cases, the difference in the expression of other genes in the presence of RSV was not significant. The exceptions are shown in Supplementary Table S2.

#### 3.2.3. The Effect of RSV

Generally, the addition of RSV under N or R conditions did not lead to a significant difference in the expressions of *SIRT1*, *SIRT3*, *PGC-1α*, *Nrf1*, and *Tfam* in both patients and controls (Table 6 and Figure 3).

## 4. Discussion

Resveratrol is believed to mimic the physiological effects of CR in a mainly SIRT1- or SIRT3-dependent manner [22,23]. Functional mitochondria have been reported to be important for the effects of RSV [24]. Thus, in the present study, this potential effect of RSV was evaluated in oxidative phosphorylation capacities and transcription factors involved in mitochondrial biogenesis in myoblasts of five patients harboring the m.3243A>G point mutation and five controls. Furthermore, it was assessed whether mitochondrial dysfunction based on an mtDNA defect in patients could trigger cellular signals provoking compensatory adaptations.

Analyzing the mitochondrial activity was performed using a Seahorse XF96 Cell Analyzer. In patients, there was no effect resulting from the addition of RSV under the N condition. Under this condition (glucose as substrate), independent of RSV-absence or presence, the important respiratory factors BR and ATP-R were higher in the controls than in the patients. This is consistent with another study reporting reduced ATP-linked respiration, MR, and overall, a decrease in mitochondrial function in fibroblasts of MELAS patients. [25]. In the R group, the medium was only used in concentrations that are necessary to fulfil the cellular basic needs, including CR, as well as lacking of several other factors, including insulin. These restrictions generally led to a decrease of respiratory values in both patients and controls compared to normal conditions. The presented findings are partly in contrast to data from previous studies, which reported an increase of the mitochondrial ATP synthesis efficiency and oxidative metabolism resulting from CR conditions [26,27,28]. The different experimental conditions or different species might be the reason for the contradicting results. In a study using C2C12 myoblasts, the measurements were performed in three groups; assaying oöconditions identical to culture conditions, with 1 g/L or without glucose. Considering the results upon assaying with 1 g/l glucose, they report a slightly higher basal mitochondrial respiration and ATP turnover-driven respiration in groups with glucose in culture medium compared to those with glucose depletion [26]. Other studies performed the experiments in mice and evaluated the CR effect by subsequent measurements in tissues. Their results should be considered as a reaction of several organs involved [27,28]. Moreover, in the present study, the effects seen in the R groups resulted from a reduction, not only of glucose but of other supplements as well, compared to the normal medium.

The acquired data under normal conditions did not confirm the reported positive effect of RSV on OXPHOS values [15,29]. Low doses of RSV have been reported to ameliorate the mitochondrial respiratory dysfunction in fibroblasts of patients carrying homoplasmic mtDNA mutations [30]. However, there are other studies reporting either no effect or a detrimental effect of RSV on ATP production in fibroblasts of controls or patients with mitochondrial disorders [31]. It has been suggested that the therapeutic effects of RSV for the treatment of mitochondrial disorders might depend on many factors, including the severity of the underlying defect and the administered dose. RSV might be beneficial to some patients as a supportive therapeutic supplement and as part of a multi-component therapy [32]. Under restricted conditions, OXPHOS values improved in both patients and controls in the presence of RSV (especially 10µM). In this situation, the above-mentioned respiratory factors were in general similar in patients and controls (Figure 2 and Table 4).

The lacking positive effect of RSV under normal conditions is consistent with a study on C2C12 cells. The addition of RSV for 24 h at a concentration of 1 µM to 10 µM did not affect the ATP production but led to a 50% decrease in ATP concentration in the 20 µM RSV group [33].

Other studies showed an inhibitory effect of RSV on mitochondrial F0F1-ATPase activity in a concentration-dependent manner in rat brain and liver mitochondria, suggesting that RSV can also impair mitochondrial metabolic pathways [34,35]. In the present study, RSV was used at 10 µM and 20 µM because even higher concentrations of RSV have been known to be lethal to cells [33].

For the evaluation of the cellular response resulting from impaired OXPHOS in patients, the relative expression of the key genes related to energy metabolism and mitochondrial function *SIRT1*, *SIRT3*, *PGC-1α*, *Nrf1*, and *Tfam* were investigated. While one study showed comparable expression of *Nrf1* and *Tfam* and upregulation of *PGC-1α* and *SIRT3* in MELAS patients compared to controls [25], another one reported similar expression rates of *PGC-1α* and upregulation of *Tfam* in patients compared with that of controls [36]. In the present study, the expression of the above-mentioned genes was similar in patients and controls; however, the *PGC-1α* values were only higher under R conditions in controls compared to patients.

There are some tissue-specific metabolic pathways to maintain energy and nutrient homeostasis in mammals, acting as a response to environmental and nutritional conditions. Fasting induces *PGC-1α* deacetylation by *SIRT1* in skeletal muscle [37]. An increase in *SIRT3* and *SIRT1* protein level and expression in skeletal muscle of mice has been reported by fasting, correlated with an induction of *PGC-1α* as well. Resveratrol, in contrast, induced the *SIRT1* expression in mice skeletal muscle but did not affect the *SIRT3* level. The inability of resveratrol to induce *SIRT3* has been interpreted as an ineffectiveness of resveratrol to mimic CR-mediated health benefits [23,38]. In the present study, the restricted condition led to upregulation of *SIRT3*, *PGC-1α*, and *Nrf1* in both patients and controls. *PGC-1α*’s upregulation was particularly pronounced in controls under R conditions (about 60× higher than under N condition). However, *Tfam*-expression was not affected upon restricted conditions. Notably, the addition of 10 µM RSV, under restricted conditions, led to downregulation of *Tfam*-expression in both patients and controls.

The restricted condition led to an increased expression of the investigated genes, which might indicate stimulation of mitochondrial biogenesis. On the other hand, respiratory key parameters were decreased under R conditions. The lower maximal capacity might either result from decreased substrate availability or a comprised mitochondrial mass/integrity or a mitophagic turnover under stressful situations to prevent accumulation of additional damage [39,40]. The studied genes showed slight upregulation in the presence of 10 µM RSV under N conditions, and upregulation in the presence of 20 µM RSV under R conditions compared to the conditions without RSV; however, not always significant (Table 6 and Figure 3).

### Limitations

Due to lacking several factors in the culturing of the restricted group, the obtained results cannot be seen as a pure effect of glucose depletion, and a direct comparison of N and R groups is not easy to establish. Further studies are necessary to investigate alternative pathways and individual factors that are influenced by RSV in stressed models. Moreover, assay conditions (N or R) were similar for cells from both conditions. An adjustment to specific experimental conditions might be considered in future works. It should be noticed that mRNA quantification does not always represent the expressed amount of protein or changed modification of these proteins as deacetylation, phosphorylation, or methylation. These factors could play a role in protein translation and, subsequently the number of active proteins.

## 5. Conclusions

The data in the present study confirmed the reduced mitochondrial respiration in patients harboring the m.3243A>G mutation. The fasting stimulating effect of RSV in myoblasts under normal conditions was not demonstrated. Interestingly, under restricted conditions, there was an improvement in ATP-linked respiration, resulting from RSV in both patients and controls. It might show that benefits of RSV occur only in stressed models. The positive effect of RSV was not always concomitant with an increase in the expression of the investigated transcription factors involved in mitochondrial biogenesis in this study.

## Figures and Tables

**Table 1:** Sex, age, and location of muscle biopsy of five patients with the genetically confirmed m.3243A>G mutation and five healthy controls, F: female, M: male.

	Gender	Age at Biopsy	Location of Muscle Biopsy
**Patients**			
P 1	M	43	biceps brachii muscle
P 2	M	42	biceps brachii muscle
P 3	F	70	quadriceps muscle
P 4	M	34	deltoideus muscle
P 5	F	40	biceps brachii muscle
**Controls**			
C1	F	50	biceps brachii muscle
C 2	M	53	quadriceps muscle
C 3	F	40	quadriceps muscle
C 4	M	35	biceps brachii muscle
C 5	F	49	biceps brachii muscle

**Table 2:** Primers used for quantitative RT-PCR.

Target Gene	Forward Primer	Reverse Primer
*SIRT1*	AGAAGAACCCATGGAGGATG	TCATCTCCATCAGTCCCAAA
*SIRT3*	CAGCAGTACGATCTCCCGTA	GAAGCAGCCGGAGAAAGTAG
*PGC-1α*	GTCCAGGCAGGAGCTTTTAGA	AGCTTTGATTTGCTCAAGCCAT
*Nrf1*	AGGAACACGGAGTGACCCAA	TATGCTCGGTGTAAGTAGCCA
*Tfam*	ATGGCGTTTCTCCGAAGCAT	TCCGCCCTATAAGCATCTTGA
*HPRT1*	ACCAGTCAACAGGGGACATAA	CTTCGTGGGGTCCTTTTCACC
*β-Actin*	GCGCCGTTCCGAAAGTTG	CGCGCCGCTGGGTTTTATAG

**Table 3:** Comparison of the mean values of the key parameters for mitochondrial function (basal, MR, ATP production and SRC) using a Seahorse XF96 Cell Analyzer in myoblasts between patients (n = 5) and controls (n = 5) under normal (N) or restricted (R) conditions. *p* values are only shown in the case of significant differences between patients and controls. −RSV = without RSV.

	**N conditions**								
	**−** **RSV**			**10 µM RSV**			**20 µM RSV**		
	Controls (mean)	Patients (mean)	*p* value	Controls (mean)	Patients (mean)	*p* value	Controls (mean)	Patients (mean)	*p* value
Basal	59.24	41.71	0.0005	56.11	39.19	<0.0001	45.44	35.44	0.05
MR	212.3	153.3	0.05	191.3	169.2		190.4	164	
SRC	156.4	144.1		135.2	115.8		137.3	116.8	
ATP	45.64	33.51	0.01	40.39	28.82	0.001	37.24	29.4	0.03
	**R conditions**								
	**−RSV**			**10 µM RSV**			**20 µM RSV**		
	Controls (mean)	Patients (mean)	*p* value	Controls (mean)	Patients (mean)	*p* value	Controls (mean)	Patients (mean)	*p* value
Basal	14.19	16.52		18.91	23.8		24.84	21	
MR	78.04	66.87		87.21	81.92		103.8	71.5	
SRC	57.88	50.34		68.79	62.84		79.22	48.38	
ATP	10.67	12.45		18.9	23.8		24.84	21	

**Table 4:** Comparison of the effect of 10 or 20 µM RSV on basal, MR, ATP production, and SRC measured under normal (N) or restricted (R) conditions in patients (n = 5) and controls (n = 5). *p* values are only shown in the case of a significant difference between the two conditions.

	**Controls**									
	**N**					**R**				
	−RSV	10	*p* (10)	20	*p* (20)	−RSV	10	*p* (10)	20	*p* (20)
Basal	59.24	56.11		45.44	0.02	14.19	18.91		24.84	0.008
MR	212.3	191.3		190.4		78.04	87.21		103.8	
SRC	156.4	135.2		137.3		57.88	68.79		79.22	
ATP	45.64	40.39		37.24		10.67	18.9	0.03	24.84	<0.0001
	**Patients**									
	**N**					**R**				
	−RSV	10	*p* (10)	20	*p* (20)	−RSV	10	*p* (10)	20	*p* (20)
Basal	41.71	39.19		35.44		16.52	23.8	0.05	21	
MR	153.3	169.2		164		66.87	81.92		71.5	
SRC	144.1	115.8		116.8		50.34	62.84		48.38	
ATP	33.51	28.82		29.4		12.45	23.8	<0.0001	21	0.005

**Table 5:** Comparison of the relative expression rate of the genes *SIRT1*, *SIRT3*, *PGC-1α*, *Nrf1*, and *Tfam* measured under N or R conditions in patients (n = 5) and controls (n = 5). *p* values are only shown in the case of significant differences between patients and controls. −RSV = without RSV.

	**Controls**								
	**−RSV**			**10 µM RSV**			**20 µM RSV**		
	N (mean)	R (mean)	*p* value	N (mean)	R (mean)	*p* value	N (mean)	R(mean)	*p* value
SIRT1	0.56	2.04		2.65	0.82		0.84	7.1	<0.0001
SIRT3	0.4	2.1	0.02	1.77	1.47		1.07	3.46	0.0003
PGC-1α	0.3	17.8	<0.0001	1.41	8.3	<0.0001	0.45	19.97	<0.0001
Nrf1	0.57	1.87		2.68	0.77	0.007	1.16	3.51	0.0004
Tfam	2.99	4.45		12.44	2.67	<0.0001	4.96	7.55	
	**Patients**								
	**−RSV**			**10 µM RSV**			**20 µM RSV**		
	N (mean)	R (mean)	*p* value	N (mean)	R (mean)	*p* value	N (mean)	R (mean)	*p* value
SIRT1	1.26	3.19		2.463	0.95		0.67	5.82	<0.0001
SIRT3	0.77	2.9	0.0009	1.49	1.36		0.57	1.45	
PGC-1α	0.27	4.7	0.006	0.94	3.78		0.54	4.43	0.02
Nrf1	0.93	2.97	0.003	1.21	0.79		0.9	3.7	<0.0001
Tfam	2.71	4.4		8.34	2.27	0.0003	1.53	2.7	

**Table 6:** The effect of 10 or 20 µM RSV on the relative expression rate of the genes *SIRT1*, *SIRT3*, *PGC-1α*, *Nrf1*, and *Tfam* measured under normal (N) or restricted (R) conditions in patients (n = 5) and controls (n = 5). *p* values are only shown in the case of significant differences between the two conditions.

	**Controls**									
	**N**					**R**				
	−RSV	10	*p* (10)	20	*p* (20)	−RSV	10	*p* (10)	20	*p* (20)
SIRT1	0.56	2.65		0.84		2.04	0.82		7.1	<0.001
SIRT3	0.4	1.77		1.07		2.1	1.47		3.46	
PGC-1α	0.3	1.41		0.45		17.8	8.3	<0.0001	19.97	
Nrf1	0.57	2.68	0.002	1.16		1.87	0.77		3.51	0.03
Tfam	2.99	12.44	<0.0001	4.96		4.45	2.67		7.55	
	**Patients**									
	**N**					**R**				
	−RSV	10	*p* (10)	20	*p* (20)	−RSV	10	*p* (10)	20	*p* (20)
SIRT1	1.26	2.463		0.67		3.19	0.95		5.82	0.02
SIRT3	0.77	1.49		0.57		2.9	1.36		1.45	
PGC-1α	0.27	0.94		0.54		4.7	3.78		4.43	
Nrf1	0.93	1.21		0.9		2.97	0.79	0.004	3.7	
Tfam	2.71	8.34	0.004	1.53		4.4	2.27		2.7	

## Supplementary Materials

The following are available online at https://www.mdpi.com/2218-273X/10/8/1103/s1, Table S1: Comparison of the mean values of basal, maximal respiration (MR), ATP production, and spare respiratory capacity (SRC) measured under normal (N) or restricted (R) conditions in patients (n = 5) and controls (n = 5), Table S2: Relative expression rates of the genes *SIRT1*, *SIRT3*, *PGC-1α*, *Nrf1*, and *Tfam* in myoblasts between patients (n = 5) and controls (n = 5) under normal (N) or restricted (R) conditions.
