# Supplementary material for: The Effect of Resveratrol on Mitochondrial Function in Myoblasts of Patients with the Common m.3243A>G Mutation
Source: Biomolecules. 2020 Jul 24;10(8):1103. doi: 10.3390/biom10081103 (PMC7464358; doi:10.3390/biom10081103)
Supplement: Supplementary file 1 [file biomolecules-10-01103-s001.pdf]

Supplementary Materials:

The effect of resveratrol on mitochondrial function in Myoblasts of patients with the common m.3243A>G mutation

Leila Motlagh Scholle, , Helena Schieffers, SamiyaAl-Robaiy, Annemarie Thaele, Faramarz Dehghani, Diana Lehmann Urban, and Stephan Zierz

Table 1. Comparison of the mean values of basal (BR), maximal respiration (MR), ATP production and spare respiratory capacity (SRC) measured under normal (N) or restricted (R) conditions in patients (n=5) and controls (n=5). *p* values are only shown in case of significant differences between two conditions. -RSV = without RSV.

Controls

	**-RSV**			**10 μM RSV**			**20 μM RSV**		
	N (mean)	R (mean)	*p* value	N (mean)	R (mean)	*p* value	N (mean)	R (mean)	*p* value
Basal	59.24	14.19	<0.0001	56.11	18.91	<0.0001	45.44	24.84	<0.0001
MR	212.3	78.04	<0.0001	191.3	87.21	<0.0001	190.4	103.8	<0.0001
SRC	156.4	57.88	<0.0001	135.2	68.79	0.0006	137.3	79.22	<0.01
ATP	45.64	10.67	<0.0001	40.39	18.9	<0.0001	37.24	24.84	0.0005

Patients

	**-RSV**			**10μM RSV**			**20μM RSV**		
	N (mean)	R (mean)	*p* value	N (mean)	R (mean)	*p* value	N (mean)	R (mean)	*p* value
Basal	41.71	16.52	<0.0001	39.19	23.8	<0.0001	35.44	21	0.0004
MR	153.3	66.87	<0.0001	169.2	81.92	<0.0001	164	71.5	<0.0001
SRC	144.1	50.34	<0.0001	115.8	62.84	0.02	116.8	48.38	<0.0001
ATP	33.51	12.45	<0.0001	28.82	23.8		29.4	21	0.02

Table 2. Relative expression rates of the genes *SIRT1, SIRT3, PGC-1α, Nrf1* and *Tfam* in myoblasts between patients (n=5) and controls (n=5) under normal (N) or restricted (R) conditions. *p* values are only shown in case of significant difference between patients and controls. -RSV = without RSV.

N conditions

	**-RSV**			**10μM RSV**			**20μM RSV**		
	Controls (mean)	Patients (mean)	*p* value	Controls (mean)	Patients (mean)	*p* value	Controls (mean)	Patients (mean)	*p* value
SIRT1	0.56	1.26		2.65	2.463		0.84	0.67	
SIRT3	0.4	0.77		1.77	1.49		1.07	0.57	
PGC-1α	0.3	0.27		1.41	0.94		0.45	0.54	
Nrf1	0.57	0.93		2.68	1.21	0.04	1.16	0.9	
Tfam	2.99	2.71		12.44	8.34	0.01	4.96	1.53	0.03

R conditions

	**-RSV**			**10μM RSV**			**20μM RSV**		
	Controls (mean)	Patients (mean)	*p* value	Controls (mean)	Patients (mean)	*p* value	Controls (mean)	Patients (mean)	*p* value
SIRT1	2.04	3.19		0.82	0.95		7.1	5.82	
SIRT3	2.1	2.9		1.47	1.36		3.46	1.45	0.003
PGC-1α	17.8	4.7	<0.0001	8.3	3.78	0.005	19.97	4.43	<0.0001
Nrf1	1.87	2.97		0.77	0.79		3.51	3.7	
Tfam	4.45	4.4		2.67	2.27		7.55	2.7	0.002
